# Detecting Antigen-Specific T Cell Responses: From Bulk Populations to Single Cells

**DOI:** 10.3390/ijms160818878

**Published:** 2015-08-12

**Authors:** Chansavath Phetsouphanh, John James Zaunders, Anthony Dominic Kelleher

**Affiliations:** 1 Kirby Institute, University of New South Wales, 2031 Sydney, Australia; E-Mails: jzaunders@kirby.unsw.edu.au (J.J.Z.); akelleher@kirby.unsw.edu.au (A.D.K.); 2 Centre for Applied Medical Research, St. Vincent’s Hospital, 2010 Sydney, Australia

**Keywords:** antigen-specific T cells, digital PCR, microfluidics, mingle-cell RNA-seq

## Abstract

A new generation of sensitive T cell-based assays facilitates the direct quantitation and characterization of antigen-specific T cell responses. Single-cell analyses have focused on measuring the quality and breadth of a response. Accumulating data from these studies demonstrate that there is considerable, previously-unrecognized, heterogeneity. Standard assays, such as the ICS, are often insufficient for characterization of rare subsets of cells. Enhanced flow cytometry with imaging capabilities enables the determination of cell morphology, as well as the spatial localization of the protein molecules within a single cell. Advances in both microfluidics and digital PCR have improved the efficiency of single-cell sorting and allowed multiplexed gene detection at the single-cell level. Delving further into the transcriptome of single-cells using RNA-seq is likely to reveal the fine-specificity of cellular events such as alternative splicing (*i.e.*, splice variants) and allele-specific expression, and will also define the roles of new genes. Finally, detailed analysis of clonally related antigen-specific T cells using single-cell TCR RNA-seq will provide information on pathways of differentiation of memory T cells. With these state of the art technologies the transcriptomics and genomics of Ag-specific T cells can be more definitively elucidated.

## 1. Introduction

A wide variety of assays can be used to characterize T cell responses in order to determine the state and capability of the immune system. Such studies can reveal fundamental mechanisms underlying immunity aid the design of clinical diagnostics, help develop intervention therapies, and determine signatures of effective immune responses [1]. Primarily, research on antigen (Ag)-specific CD4+ T cells has been done using bulk-sorted populations by focusing on small sets of cells defined by selected markers that were hypothesized to identify homogenous sub-populations; for example, central *versus* effector memory cells defined by surface markers such as CD45RA/RO isoforms and CCR7 or CD62L expression [2,3,4]. However, accumulating data demonstrate that there is considerable heterogeneity within the Ag-specific population on the basis of genomic differences, cytokine secretion profiles, function, and trafficking markers [1,5,6,7,8].

Standard analytical technologies historically have measured the average response from highly heterogeneous populations. Common assays that detected cell proliferation, cytolytic activity, and cytokine expression have yielded valuable insights into disease pathogenesis and immunity to microbes such as viruses and tumour or self-antigens. However, these assays examined multiple parameters at the population level, where the implicit averaging of many measurements may mask the specific involvement of individual cells and the interactions that can occur between neighbouring cells. These technologies made it difficult to infer the characteristics of rare subsets of cells, such as Ag-specific T cell responses, without first purifying subsets of T cells. Even when purified, the T cell subsets were generally identified on the basis of a relatively small number of markers, compared to the much larger number of cell surface proteins expressed by T cells [9,10,11]. Single cell analyses are beginning to show that these approaches have underestimated heterogeneity.

Recently, single-cell analyses have focused on measuring the quality and breadth of a response. Variations in the expression of molecules between individual cells are thought to play an important role in functionally diversifying an immune response at the population level and also determining the diverse anatomical locations of individual cells. Advances in genome-wide quantitative analysis of single cells can provide an important vehicle that allows the investigator to make further insights into the variation between individual cells and to determine how these impact on the fine specificity of the nature and regulation of the immune response.

The challenge of understanding heterogeneity between cells, particularly tumour cells [12,13,14] has driven many of the major technological advances, resulting in the design of powerful instruments, protocols, and analysis protocols that enable the elucidation of DNA, RNA, and protein expression at the single-cell level. Flow cytometry has been widely adopted as the cornerstone of high-throughput analysis of specific protein expression and phosphorylation states of single cells within complex populations. Cell sorting has typically been used to purify up to six populations at a time from these mixtures of cells. The recent coupling of this technology with microfluidics and genome-wide deep sequencing at the single cell level has enabled further insights into cell biology. Single-cell genomics provides the basis for unbiased investigations into the molecular and functional consequences of cellular variability. In this review, the advantages and disadvantages of standard T cell response detection assays will be discussed. Newer technologies to more comprehensively define T cell responses at the single-cell level will be examined and the advances in single-cell genomics will be highlighted.

## 2. Standard Assays Are Insufficient for the Detection of T Cell Responses at the Single Cell Level

Measuring T lymphocyte proliferation after antigenic or mitogenic stimulation is an important parameter used in diagnosis of various immuno-deficiencies and in the monitoring a variety of immune responses. Measurement of the incorporation of tritiated thymidine [^3^H] into lymphocyte DNA is a common approach used to determine the extent of antigen- or mitogen-driven cell proliferation [15]. Disadvantages of this assay include: The response of individual populations cells cannot be delineated without cell sorting; the inherent variability of the assay; the limitations and safety of handling radioactive material; and the labour-intensive nature of the protocols (*i.e.*, multiple wash steps involved, PBMC isolation, and inability to determine which cells are proliferating) [16].

More recent approaches that overcome some of these problems include the use of the cytoplasmic dyes, such as carboxyfluorescein succinimidyl ester (CFSE), to track lymphocyte proliferation [17]. CFSE covalently binds to amino groups on intracellular macromolecules that anchor the dye. CFSE is inherited equally by daughter cells after division, resulting in the halving of mean fluorescence with each generation. Disadvantages of this assay are: The time required for proliferation detection is usually 4–6 days; and high concentrations can cause cell toxicity and impair expression of activation markers [17,18]. However cell subpopulations can be tracked if markers are expressed stably. The use of multi-parameter flow cytometry allows other information about the proliferating cells and their phenotype to be accumulated. Further, the stability, or otherwise, of markers during the process of proliferation in response to antigen can be definitively determined.

ELISPOT (enzyme-linked immunospot) and intracellular cytokine staining (ICS) are widely used assays for the detection and enumeration of antigen-specific T cells. ELISPOT assays detect antigen-induced secretion of cytokines (usually IFN-γ) trapped by an immobilized antibody on a nitrocellulose membrane and visualized by an enzyme-coupled secondary antibody. ICS also detects cytokines produced by antigen-stimulated T cells, via the detection of the cytokines, such as IFN-γ, TNF, and IL-2, trapped within golgi/ER bodies in the cytoplasm through inactivation of granule secretion by brefeldin A or monensin, and visualized via fluorochrome-labelled monoclonal antibodies (mAb) after cell permeabilisation followed by flow cytometry.

The use of both assays is associated with implicit *a priori* assumptions regarding the importance of certain cytokines in the responses of interest (*i.e.*, IFN-γ) and do not take into account the other aspects of the T cell response; most often used to measure Th1 or Th2 responses, but can also be used to measure effector molecules such as Granzyme B. ELISPOT can only measure one or two parameters at a time and, without depletion or purification of cell subsets, the source of the cytokine cannot be determined. Multiple parameters in the ICS need to be optimized for each model system, such as stimulation, fixation/permeabilization, and appropriate controls need to be considered [19]. These assays are typically restricted to Th1 subset identification, as Th2 cytokine detection works poorly in these assays, and Th17 responses are usually only detectable when mitogens are used. This is due to a combination of factors including limitations in availability of mAb and the lower levels of cytokine production. Further, these assays do not allow live isolation of cells for downstream functional or molecular analysis and bulk populations with high cell numbers are required for each assay [20,21].

Detailed information of responding single cells in ELISPOT assays is limited to the level of markers used to purify cells prior to the assay, while ICS assays are limited by the number of additional fluorescence markers that can added to the assay. Each assay comes with issues of assay validation and quality control, with advantages and disadvantages depending on the nature of the responses detected. For example, standardization of assay requires the removal of differences in confounding variables when comparing experiments performed by the same or different users under the same conditions. Issues, such as choice of starting material, coating techniques, incubation and washing steps, and personal preference in reading spot development, need to be taken into account [22]. Additionally, currently-available flow cytometric analysis is generally limited to a maximum of 16 fluorochromes, but the use of up to 29 parameters has been recently reported [1,23,24]. The use of mAbs labeled with rare-earth isotopes, combining flow cytometric and mass cytometric technologies (CyTOF^®^), also greatly increases the number of parameters able to be defined on each cell [25,26]. A caveat with the CyTOF^®^ platform is that it still lacks the sensitivity of standard fluorescences cytometry, up to three-fold less sensitive and requires more cells for staining. Nevertheless, the available number of parameters still does not approach the number of proteins that are variably expressed by T cells [27,28].

### 2.1. Using MHC-Multimers to Identify Antigen-Specific T Cells

Recombinant multimeric complexes of soluble recombinant MHC molecules often referred to as “tetramers” have emerged as a key tool for elucidation of the frequency of antigen-specific T cells *in vitro*, particularly in viral infections and post-vaccinations. Since 1996, there has been a revolution in the characterization of antigen-specific T cells, due to the development of reagents consisting of soluble multimerized MHC-I peptide complexes to detect epitope specific CD8+ T cells using flow cytometry through their increased avidity for TCR [29,30]. MHC-I tetramers combined with other staining techniques have been used to examine detailed information about antigen specific T cells, such as levels of activation, effector function, proliferation, and apoptosis [31], but importantly because they bind to the surface of T cells based only on their expression of TCR which recognize the incorporated epitope cells which are anergic, “stunned” or “exhausted” can be identified by this technology [32,33,34,35,36]. Further, because only surface staining is required, the cells can be sorted and used for functional assays and/or RNA profiling. This has led to the colossal expansion of information on antigen-specific CD8+ T cells and to a lesser extent antigen-specific CD4+ T cells, because of the more limited availability of MHC-II tetramers [37].

MHC-I-peptide complexes consist of an invariant light chain (β_2_-microglobulin), a polymorphic heavy chain and a specific 8–10 amino acid peptide. The presence of a cognate peptide in the antigen-binding groove is essential for the formation of MHC-I molecules. This association is based on specific complimentary interactions between amino acid side chains at the anchor positions of the peptide and allele-specific pockets [38]. This helps ensure that the recombinant MHC-I heterodimers that refold are conformationally correct. Typically the formation of these soluble MHC-I multimers is dependent upon the specific biotinylation of a tail engineered to replace the transmembrane and cytoplasmic domains of the heavy chain. The biotinylated heterodimers are then bound to each of the four binding sites of fluorescently labeled streptavidin, forming a tetrameric complex. However, other strategies for multimeriziation have been employed to achieve the necessary increase in avidity between MHC-I and TCR to allow interactions stable enough for robust staining of T cells and subsequent identification by flow cytometry [39,40,41,42]. A possible drawback from this technique is that individual tetramers have to be designed for each epitope of interest, and then are only applicable to individuals carrying a particular class I allele. The shelf life of the constructs are variable, and positive and negative controls need to be carefully identified to ensure staining is specific, thus making the process very labour intensive, especially if multiple epitope-specific responses are to be studied simultaneously [37]. To overcome these limitations, dextramers (multimers with a dextran backbone) were developed, each dextramer molecule bears multiple fluorescein and streptavidin components. These dextramers were able to identify low frequency antigen-specific T cells and produced stronger signals than their tetramer counterpart [43].

In contrast to the relative ease of production MHC class I tetramers, the development of MHC class II tetramers has been more difficult. There are multiple reasons for this. Firstly, the peptide-binding grove depends on correct association of variable alpha and beta chains making the synthesis of some alleles inefficient. The characteristics determining binding of antigenic peptide to MHC-II are different to MHC-I in several ways. Secondly, the stability of the class II molecule is not dependent on the binding of peptide in the groove. The groove is open-ended at either end allowing longer peptides to bind and these may bind in different registers within the groove. Further, efficient loading of peptide occurs at acid pH, is dependent upon molecules such as HLA-DM and DO catalysing the efficient exchange of peptide for the CLIP peptide and the peptide receptive state of the class II molecule is rapidly lost. These conditions are difficult to reproduce *in vitro*. In addition, there is much greater promiscuity of peptide binding to class II than class I, which has resulted in much lower rates of definitive description of class II restricted epitopes [44,45]. Many methods have been explored in an attempt solve these problems. One of these methods is producing class II proteins by co-expressing the α and β subunits in mammalian or insect cells and relying on *in vivo*, rather than *in vitro*, re-folding, as is the case with proteins produced from *E. coli* [44]. Unfortunately, much of the material bound to the binding groove is not the epitope of interest and the exchange of peptide into the binding groove is often inefficient. To overcome this constructs expressing a fusion protein, consisting of peptide fused to MHC-II β N-terminus via a flexible linker region have been trialled. This covalently attached peptide has preferential access to the peptide-binding site, thus increasing the rate of the correct peptide occupying the binding groove [46,47]. Other modifications used to enhance the assembly of the subunits have included the use of leucine zippers and chimeric IgG Fc domains to promote assembly and stability of the heterodimer [48,49]. Combined these approaches have resulted in a slow, but steady, increase in the availability of the pool of reliable MHC-II multimers, though their availability is still far more limited than MHC-I equivalents. With these methods of MHC-II production, it is now theoretically possible to identify a subset of antigen-specific CD4+ T cells with multimeric complexes [37,50]. This will facilitate the isolation of single antigen-specific CD4+ T cells for downstream analysis [51,52]. MHC/peptide tetramers, termed streptamers, were developed for this purpose. Streptamers can be reversibly dissociated from binding to antigen-specific CD4+ T cells, this feature allows these cells to remain functionally active, contrary to conventional tetramers that impairs lytic function and proliferation of the bound cells [53].

### 2.2. Cell Surface Detection of Antigen-Specific T Cells

Apart from ICS and multimeric MHC-II complexes, other approaches have been reported to use flow cytometry to identify Ag-specific T cells, including cell surface trapping of cytokines using magnetic bead technology (Miltenyi cytokine-capture system^®^) [54], or the use of activation-induced trafficking of some intracellular markers to the cell surface such as CD40L expressed on CD4+ T cells [55,56]. However, both these assays are limited by their detection of only one effector molecule at a time and we know that many important responses, such as Tregs and CTL, are not detected by either of these methods.

### 2.3. CD25/OX40 Assay

CD4+ T cell responses are pivotal to the regulation of the immune system during viral infection. Due in great part to the difficulties associated with the synthesis and use of class II multimers and therefore an inability to identify and isolate epitope specific CD4+ T cells, our understanding of the molecular basis of CD4+ function has fallen behind that of CD8+ T cells. Thus an assay that allows live isolation of antigen specific T cells is necessary.

A recent flow cytometric assay, developed by Zaunders *et al.*, employs the co-expression of CD25 (IL-2Rα) and CD134/OX40 (a TNF receptor family member) to identify antigen-specific CD4+ T cells. The co-expression of these two molecules in CD4+ T cells is very low in peripheral blood. However, with antigenic stimulation *in vitro*, their expression becomes up regulated over time, peaking at approximately 44 h post-stimulation. Advantages of this assay are that it detects a more global population of antigen-specific cells that appear to include all Th1, Th2, Th17, Tfh, and Treg lineages and is, therefore, not biased by detection of a particular cytokine or effector molecule, resulting in higher levels of antigen-specific T cells than ICC and ELISPOT. Further, it allows live isolation of individual cells for downstream functional and molecular characterization [7,57,58,59,60,61,62].

## 3. Enhanced Flow Cytometry Techniques for Single-Cell Analysis

Flow cytometry has been extensively used to analyse protein expression within cells. The relatively recent development combining microscopic imaging and fluorescence-activated flow cytometry has allowed a more in-depth examination of single cells. Each cell can be examined for morphology, as well as the spatial localization of the protein molecules within the cell. Instruments, such as the Imagestream^®^, allow rapid acquisition and processing, enabling measurements of thousands of cells per second, which is an advantage over conventional microscopy platforms [63]. This combination technology has been useful for the determination of protein localization, cell morphology, observation of protein interactions during signalling cascades and cellular uptake of foreign particles [64,65]. Although highly useful for providing information on single cells, the measurements acquired are only obtained at a single snapshot in time.

Newer technologies that complement and augment the data generated from flow cytometry have been developed. These technologies can assess the functional and transcriptional dynamics of cells at the microliter to picolitre scale. The two types of techniques that have emerged as tools to detect immune responses are microfluidic systems and spatial arrays with nanolitre-scale wells. For a detailed review on nanolitre micro-well and micro-dense arrays please refer to a review by Lindstrom, *et al.* [66].

Micro-dense arrays contain up to 100,000 sub-nanolitre welled compartments that allow the isolation or distribution of single-cells and measurement of cellular function, protein secretion and mRNA in parallel. This platform provides the ability to examine cellular interaction between different cell types and provides highly-specific resolution not found when using bulk populations. These cells can be deposited into nanowells for co-culture by dispensing each cell onto the array sequentially and allowing cells to settle via gravity, these cells can then be used for further analyses [1]. Co-cultures of cells (e.g., one effector cell and one antigen-presenting cell) in individual wells can provide insight into intercellular signaling and interaction dynamics. This technology can be used to assess antigen-specific interactions between cognate T cells and their corresponding antigen presenting cell to detect activation, cytolysis and cytokine production [67]. This approach has demonstrated that HIV-specific CD8+ T cells responses initially involve either cytolysis or secretion of pro-inflammatory cytokines (*i.e.*, IFNγ) [68,69].

Nanolitre micro-well arrays enable the study of single-cell phenotyping of rare cells, such as antigen-specific T cells and B cells. Studies on these rare subsets have revealed structure-function relationships between molecular synapses and signalling cascades [70]. Co-culture studies together with barcoded antibody arrays enabled the examination of the influence of paracrine signalling molecules on tumour cell function and signalling networks via the multiplexed detection of both intracellular and secreted proteins/cytokines [71,72]. These nano-arrays have also been used to examine serial killing capacities of Natural Killer (NK) cells, whereby NK cells were able to kill MHC-I deficient tumour cells and also showed that simultaneous interaction with several target cells increases the cytotoxic responsiveness of NK cells [73]. This platform has been used to show to the extent of variability among homogeneous populations. Dura *et al.* investigated the heterogeneity of CD8 T cells upon antigen presentation and correlated this with early activation events. They discovered that the cells showed relatively homogeneous calcium mobilization with high antigen stimuli with uniform timings of activation. However, the response pattern became more heterogeneous with lower antigen concentrations. These cell populations could be grouped into distinct clusters. Measurement of early signalling events simultaneously revealed high heterogeneity in ERK phosphorylation in single-cells, despite uniform timing and stimulus strength [74]. This study demonstrates the potential of micro-scale tools to clarify the complex intercellular interactions initiating and regulating T cell activation through the measurement of multiple parameters over a substantial number of individual cells. However, there are some technical limitations in this system, which include the limited control on the fluidic microenvironment required to maintain the cells in culture, as well as the risk of cross-contamination between wells during the rinsing of the array chips [75].

## 4. Microfluidics and Digital PCR at the Single-Cell Level

Reverse transcription quantitative polymerase chain reaction (RT-PCR) has been extensively used to examine gene expression patterns in T cells and has been the basis for many systems biology approaches profiling cellular activity. It provides exceptional specificity and sensitivity and has been adapted for the measurement of gene expression in single cells [7,76]. The challenges of single-cell RT-PCR studies include the cumbersome and laborious steps in purifying mRNA from individual cells, and the difficulty in synthesizing and purifying cDNA from single cells [77]. These difficulties arise from the loss of starting material during cell isolation, lysis, and cDNA synthesis steps. The loss of material may be caused by mRNA degradation, adhesion to plastics, and inefficient reverse transcription [78,79]. Microfluidic devices have been developed as platforms to overcome these particular problems and to handle the low reaction volumes required for single-cell analyses, while allowing relatively high-throughput, whereby multiple samples can be run simultaneously on the same device under standardized conditions.

One of the commercially-available valve-based microfluidic qPCR systems that have been successfully used for single cell studies is the Dynamic Array™ (Fluidigm). This is a low-volume (nanolitre) system that allows high-throughput with assessment of up to 96 parameters in 96 cells per run. It allows low copy detection while being used for large-scale studies [76]. The concordance of copy number detection between microfluidics and digital PCR compared to conventional PCR has been demonstrated [80]. This technology offers a higher level of precision and can be used to measure rare transcripts [81], as well as small RNA species (*i.e.*, microRNA) [82,83].

Mingueneau *et al.* used this platform to examine early thymocyte differentiation of αβ T cells. Gene expression patterns during early T cell differentiation were measured. It was found that during transit through the CD4+CD8+ stage, these double-positive thymocytes showed a global repression of housekeeping genes, which is rare among other cells of the immune system and correlated highly with the expression of c-Myc. They also identified genetic signatures that distinguished cells destined for positive selection *versus* apoptosis [84]. Johnson *et al.* used the Fluidigm^®^ platform to define transcriptional profiles of HIV-specific CD4+ T cells. In doing so they identified a distinct transcriptional signature of HIV-specific cytolytic CD4+ T cells compared to Th1 cells, and these signatures were similar to features found in HIV-specific CD8+ T cells. These cytolytic CD4+ T cells also showed comparable killing activity to their CD8 counterpart and worked co-operatively to destroy virally infected target cells [85].

These examples demonstrate the innovative uses of these technologies; however, only a selected number of genes can be examine within one cell at a given time. Although this technology expands on the fairly outdated capabilities of traditional PCR, the elucidation of the entire transcriptome would be too cumbersome for this platform. Separation of single cells using microfluidics and the extraction of total DNA or RNA for next-generation sequencing would solve this issue. An example of this is the Fluidigm^®^ C1 single-cell isolation instrument which allows automated cell capture for RNA/DNA extraction and nucleic acid amplification that is highly useful for RNA-seq and downstream genomic analysis. Microtools such as these allow clinical samples consisting of very small cell numbers to be examined [86]. Examination of cells from cytobrushes and biopsies allows the comparison between cell phenotypes, cell-to-cell communication [87], and function at mucosal sites, which is important in many disease conditions, such as HIV-1 infection.

## 5. Single-Cell Genomics Analysis via Next-Generation Deep Sequencing

A new and important extension of bulk transcription technologies, single-cell RNA sequencing (RNA-seq) has emerged as a powerful tool for mRNA expression analysis and allows genome-wide transcriptomics to be explored [88]. The transcriptome encompasses an essential part of cell identity and function, as RNA is essential for regulation, house-keeping, effector and messenger roles. The state of a specific single-cell (e.g., antigen-specific T cells) can be assessed via the profiling of all coding and non-coding transcripts that eventually leads to genome-wide transcriptomics analysis. RNA-seq techniques involve the conversion of cellular RNA transcripts into cDNA and subsequent sequencing in parallel by using next-generation sequencing technologies [89]. This technology enables high-resolution analysis of single-cells, whereby important cellular events such as alternative splicing (*i.e.*, splice variants) and allele-specific expression can be studied, that will also aid the discovery of new genes [90].

Single-cell RNA sequencing has been to investigate the role of *de novo* hormone synthesis in T helper cells and its role in T cell homeostasis. Mahata *et al.* demonstrated that that Th2 cells produce the steroid, pregnenolone, which inhibits T helper cell proliferation and B cell class switching. They proposed that this lympho-steroid is produced in an intrinsic manner by Th2 cells during allergic immune responses to restore immune homeostasis [91]. Shalek *et al.* used single-cell transcriptomics to assess heterogeneity of mouse bone marrow-derived dendritic cells (BMDCs) responding to lipopolysaccharides. They found bimodal variance in the RNA abundance and splicing patterns within responding cells. The observed splicing patterns displayed high levels of heterogeneity between cells. They identified 137 highly variable, but co-regulated, antiviral response genes that may be propagated through an interferon feedback loop involving the transcription factors Stat2 and Irf7 [89]. These studies highlight the promise and power of single-cell RNA-seq. This technology can be used to uncover the functional diversity between cells, as well as, discovering new gene regulation circuits.

### 5.1. Detecting Clonally-Related Ag-Specific T Cells Using TCR RNA-seq

A single-cell method to assess T cell receptor beta chain (TRBV) and alpha chain (TRAV) sequence data of sorted CD4+ and CD8+ T cells has recently been published [92]. This method also describes a method allowing simultaneous measurement of mRNA transcripts for up to 34 CD4+ T cell lineage-defining transcription factors and cytokines. Three steps of nested PCR amplification from a single cell, including barcoding from each well of up to 20 separate 96-well plates, followed by pooling and deep sequencing, allows high throughput TCR sequencing and RNA profiling in parallel in 1000 s of single cells, which will dramatically increase our ability to consider relationships between TCR repertoire and T cell phenotypic or functional subsets in a range of immune responses. Previous approaches using cloning and Sanger sequencing have not been feasible for this number of cells. Also, the combination with other genomic data on the transcription factors and cytokines, using this approach is an extremely favourable combination of cost-effectiveness and high throughput, for the first time definitively matching information on clonality and genotype. Detailed analysis of clonally-related antigen-specific T cells will finally provide information on pathways of differentiation of memory T cells not possible by other currently available techniques.

## 6. Conclusions

**Figure 1:**
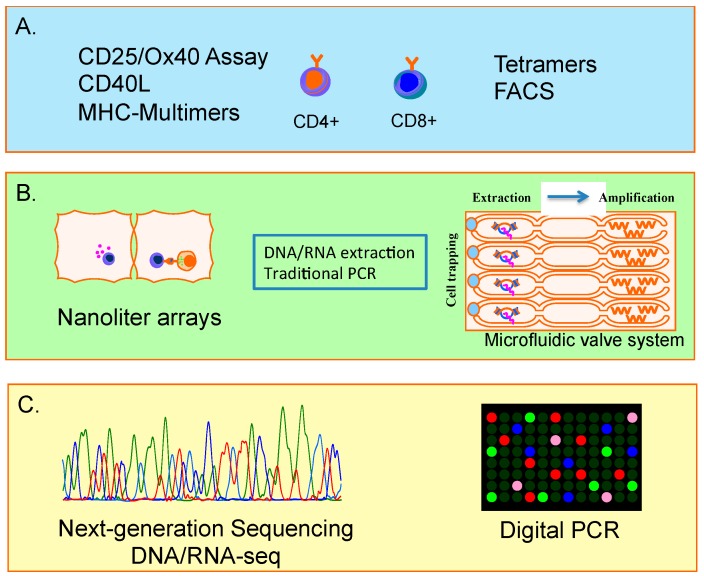
Analysis antigen-specific T cells at the single cell level pipeline. (**A**) Detection of antigen-specific CD4+ and CD8+ T cells using multiple assays and MHC-multimers; **(B**) Functional analysis of single Ag-specific cells via micro-well dense arrays and nanolitre arrays or single cell trapping using microfluidic valves for further molecular extraction/amplification; (**C**) Transcriptomic analysis using digital PCR and next-generation sequencing technologies (*i.e.*, RNA-seq) (coloured lines and dots represent fluorescence signals).

The final goal of complete genomics of the full range of single antigen-specific T cells is getting closer, due to very substantial and rapid improvements in identification of antigen-specific T cells, particularly CD4+ T cells, combined with advances in single cell mRNA technology (Figure 1). Finally, it is hoped that such studies will discriminate favourable outcomes after vaccination, or after pathogenic infection and provide a road map for rationale, rather than empiric development of vaccines and immunotherapeutics for chronic infections and drug resistant cancers.
